# The Politics of Interdictive Jurisprudence: Interrogative Practices and Psychological Evaluations (*Gemütszustandsuntersuchungen*) in the Adjudication of Civil Interdiction Cases before Berlin’s District Courts (1877–1914)

**DOI:** 10.1007/s00048-025-00430-8

**Published:** 2025-10-22

**Authors:** Eric J. Engstrom

**Affiliations:** Department of History, Humboldt University, Unter den Linden 6, 10099 Berlin, Germany

**Keywords:** Psychological evaluations, Interdiction, Civil law, Forensic psychology, Forensic psychiatry, Berlin, Gemütszustandsuntersuchungen, Entmündigung, Zivilrecht, Forensische Psychologie, Forensische Psychiatrie, Berlin

## Abstract

The article investigates the psychological evaluations (so-called *Gemütszustandsuntersuchungen*) that were used in legal interdiction proceedings at Berlin’s district courts in the late nineteenth and early twentieth centuries. The evaluations were undertaken in order to help judges decide whether or not individuals—usually, but not always feeble-minded or mentally ill ones—should be placed under legal guardianship. The following themes are addressed: the evolving procedural statutes that governed the exercise of judicial discretion and the presentation of scientific evidence; the collaborative interaction of judges and forensic experts during the interrogations; the instability of written transcripts and recourse to bodily and behavioral attributes in the face of interrogative failure; and the heated political exchanges about the psy-disciplines and their role in the abrogation or abridgment of citizens’ rights in Wilhelmine Berlin. The article will first survey the specific statutory context that framed guardianship cases in Berlin’s district court (*Amtsgericht*). It will then summarize contemporary debates about reforms to procedural law and the administrative adjudication of those cases. Against this backdrop, the analysis will then turn to an examination of court transcripts of the interrogations in order to assess the practice and often contested standing of psy-experts in the courtrooms of Wilhelmine Berlin.

## Abstract (de)

Als Beitrag zur Erforschung der forensischen Praxis und des Einflußes der sog. ‚psy-Disziplinen‘ in den Gerichtssälen des Kaiserreichs werden die Gemütszustandsuntersuchungen am Berliner Amtsgericht untersucht. Die Untersuchungen waren gesetztlich festgeschriebene Bestandteile der Beweisaufnahme im zivilrechtlichen Entmündigungsverfahren. Im Umgang mit sog. ‚Imploraten‘ (meist psychisch kranke oder an Demenz leidende Personen) entfaltete sich unter Richter, Anstalts- und Gerichtsärzte, Staatsanwälte und Protokollisten eine kollaborative Zusammenarbeit, aus der eine juristische Entscheidungen nicht über Zurechnungsfähigkeit, sondern Dispositionsfähigkeit hervorging. Die Organisation dieser gerichtlichen Untersuchungspraxis und die teils divergierende Prioritäten der beteiligten Akteure sind Gegenstand des Aufsatzes. Zunächst wird die Entwicklung der material- und prozessualrechtlichen Voraussetzungen für die Gemütszustandsuntersuchungen behandelt. Darauf folgt einen Überblick über die zeitgenössische Debatten zur Reform der Zivilprozessordnung (1877, 1898) und des Bürgerlichen Gesetzbuches (1900). Vor diesem Hintergrund werden dann die gerichtliche Untersuchungsprotokolle herangezogen, um die forensische Vernehmungspraktiken und -strategien sogenannter ‚Psy-Experten‘ am Berliner Amtsgericht vor dem Ersten Weltkrieg zu analysieren.

## Introduction

In his seminal article on “dangerous individuals,” Michel Foucault (1978: 3) envisioned civil law as an essential component in a two-stage development of the “psychiatrization of criminal danger” in the nineteenth century. The first stage relied on the notion of homicidal mania to impute insanity to those deviants whose violent behavior, because it was so monstrous and explosive, lay outside reason and comprehension. He described homicidal mania as a “point of convergence” at which jurists, who because they could discern no coherent motive for the crime were left hamstrung when apportioning punishment for it, encountered psychiatrists, who argued that such dangerous behavior was per se irrational behavior. By the end of the nineteenth century, however, this point of convergence had expanded to a “psychiatric and criminological continuum” (12) across a far wider range of infractions. Foucault attributed this shift to a growing appreciation that madness could be not just a cognitive disorder, but an emotional or hereditary one as well. Specifically, he argued that there emerged a new “psycho-sociology of delinquency” (14) that relied on notions of probability and risk that were associated with the litigation of no-fault responsibility and liability for damages in civil rather than criminal courts. Foucault described an expansion in the allocation of risk from the monstrous figure of the homicidal maniac to far more common, everyday forms of risky deviant behavior. Finally, he interpreted this transition not simply in terms of a “medicalization” of the law, but instead of a “perpetual mechanism of summoning and of interacting between medical or psychological knowledge and the judicial institution” (17) which generated its own objects and practices.

This article turns its attention to these objects and practices and how they arose out of the exchanges between psychiatrists and civil law jurists as they went about adjudicating interdiction cases. It does so within an historiographic domain, however, where relatively little attention has heretofore been paid to civil law. In civil law, experts were commonly called upon to address disputes involving wills, business and marriage contracts, and liability for damages. Above all, however, they lent their expertise to the adjudication of interdiction cases (*Entmündigungen*) and the placement of individuals under guardianship. And yet, to judge by the existing literature, nothing of much forensic significance happened in civil litigation. Instead, historical research on forensic expertise in the psy-disciplines in Germany has focused largely on questions about the reliability of witness testimony, or about the culpability of mentally ill defendants in criminal trials.^1^ Several studies have also analyzed the work of forensic experts in assessing cases of traumatic neurosis, especially those involving industrial accidents or the compensation claims of World War I veterans.^2^ Compared to these studies of forensic interventions in the fields of criminal and social-insurance law, far less interest has been shown in the influence of psychiatric and psychological expertise in civil law, although arguably its impact there, as Foucault suggests, has been equally consequential.

As a result of this historiographic oversight, much of the cultural work performed in civil courts—the identifying, sorting, and assessing of individuals—has remained unexamined (Blumenthal 2016: 13; Moran 2019). Seeking to redress the long-standing historiographic imbalance, this article examines the psychological evaluations (so-called *Gemütszustandsuntersuchungen*) that were used in legal interdiction proceedings at Berlin’s district courts in the late nineteenth and early twentieth centuries. These evaluations were undertaken in order to help judges decide whether the potential risk posed by an individual—usually, but not always a mentally ill one—justified stripping them of their rights and placing them under legal guardianship.

On its surface, the civil evaluation differed little from its counterpart in criminal law. Certainly, both were involved in the production of actionable truths. But while the aim in criminal proceedings was generally to establish willful intent (*Zurechnungsfähigkeit*), in civil interdiction proceedings the aim was to determine an individual’s ability to attend to their own affairs (*Dispositionsfähigkeit*). Whereas the attention of forensic experts in criminal trials usually focused on the defendant’s mental state *in the past, at the time of a crime*, in interdiction cases the focus was on the person’s immediate *present condition* and *future prospects*. And while criminal interrogations tended to rely on some form of coercion (be it physical, verbal, or psychological), civil ones hewed closer to investigative practices designed to elicit and document signs of behavioral and psychological deviance. The purpose of the proceedings was not to forcibly discover the facts of crime, but to establish whether someone suffered a mental defect and what legal consequences should flow from that determination, ostensibly to protect them and their relatives, not to punish them.^3^

Although mostly absent from histories of forensic medicine, these evaluations and their place in the larger juridical landscape of interdiction proceedings became the subject of intense debate in the late nineteenth and early twentieth centuries. Numerous scandals about the purportedly illegal incarceration of individuals in psychiatric asylums, as well as the enactments of new civil procedure laws (1877, 1898) and a new civil code (1900), helped to spur public and professional concerns about how some people came to have their civil rights abrogated and what role medical experts played in that process. These concerns generated a slew of monographs, handbook articles, and other public interventions.^4^

Examining a wide swath of this literature, as well as surviving transcripts of the civil evaluations, this article tracks what contemporary actors believed was at stake and analyzes the strategies and practices that they deployed in their work. Specifically, I address the evolving legal statutes that governed interdictive jurisprudence; the collaborative interaction of judges and forensic experts during the interrogations, the instability of written transcripts when verbal exchange degraded or became impossible; the recourse to bodily and behavioral attributes in the face of interrogative failure; and the heated professional exchanges among forensic experts about the proper course of reform.

In addressing these themes, the article pursues several specific aims. It reconstructs the statutory framework in which the psychological evaluations were put to use, and from which they derived their meaning and legitimacy. It also evaluates the relevance of the interrogation^5^ and of court transcripts as sites of jurisdictional contest in the division of forensic labor. Most of all, it explores some of the procedural practices and tactics of interdictive jurisprudence, which although not generally codified, often enough helped manage the process and shape its outcome.

For pragmatic reasons, my focus will be mainly on actors and events related to Berlin’s district courts (*Amtsgerichte*) in an effort to assess the reach and limits of psycho-medical influence in civil judicial proceedings in Wilhelmine Berlin and to contribute to a larger historiographic debate about the role of the psy-disciplines in the abrogation or abridgment of citizens’ rights.

## Statutory Contexts and Practices

### Prior to German Unification in 1870/71 (ALR, AGO)

For much of the nineteenth century in Prussia, the statutory grounds for interdiction in material law were found in the *Allgemeines Landrecht für die Preußischen Staaten* (ALR) (1794). The law defined the concepts of *Rasende, Wahnsinnige*, and *Blödsinnige* in association with an inability to reason, to understand the consequences of one’s action, or to manage one’s affairs.^6^ In Prussia, interdiction took the form of a so-called *Blödsinnigkeitserklärung*, a time-consuming judicial determination that was, among many other things, originally a *de jure* prerequisite for commitment to a mental asylum (Unger 1898: 5–6; Kuban 1997). In practice however, this legal threshold was generally circumvented by officials who increasingly had patients committed provisionally, thereby allowing interdiction proceedings to run their course (or not) after *de facto* admission. Already by the 1820s, most asylum admissions were not proceeded by interdictions; and even after admission, courts were notoriously slow to initiate proceedings (Sander 1865: 194–95). Furthermore, with the expansion of therapeutic institutions and the prospect of convalescing patients returning to their former lives after relatively short hospital stays, interdiction was gradually decoupled from institutionalization. Over time, courts and district attorneys in Berlin increasingly put off the initiation of interdiction proceedings until doctors had established patients to be incurably insane or demented (Schroeter 1882: 322; Unger 1898: 19–21). By the early 1890s any routine coupling of admission with interdiction had been effectively abandoned.

In terms of procedural law, the *Allgemeine Gerichts-Ordnung* (AGO) (1793) had set down guidelines for the adjudication of interdiction cases.^7^ Among many other things, these guidelines governed the evaluation of the mental state of interdictees (so-called *Imploraten,*^8^ that is, the feeble-minded, demented, or insane targets of the judicial proceedings) and the participation of forensic experts. Specifically, the AGO stipulated the conditions under which someone could be judged “*wahn- oder blödsinnig,*” including a “closer examination of the mental state of the interdictee” by a deputy judge and two medical experts.^9^ These general rules were soon complemented by a series of ministerial decrees that introduced anamnestic information into the transcript (*Protokoll*) and stipulated that medical experts had to examine the subject and query relatives and physicians in exploratory visits (*Explorationstermine*) prior to formal legal proceedings (*Kollegium*).^10^ The decrees furthermore reinforced the status of the transcript as equivalent to a postmortem examination report and required, if necessary, that experts later submit a comprehensive narrative justifying their preliminary assessment.

In the process of evaluating the prospective interdictees, contemporaries drew a clear distinction between exploratory visits and the formal proceedings. Each served different purposes and demanded a different set of inquisitorial skills. In terms of forensic practice, it is important to distinguish these two modes of examination. Forensic experts saw the examination *extra foro* as being conducted according to “clinical principles,” whereas the subsequent examination *in foro* wasn’t (Mendel 1906: 343, 353). One of the more concise accounts of these different skills was published in 1860 by the influential alienist Heinrich Neumann under the title *Theorie und Praxis der Blödsinnigkeitserklärung.* According to Neumann, the exploratory visit was intended to allow the expert to gather “all sorts of data,” “study the patient,” and orient himself so as to ensure that his final assessment was “precise and well-rounded.” In foro, however, performative and “experimental” skills were required so as to elicit a convincing response, so as to bring about “the mental conditions under which the patient’s mind is shown, as quickly and convincingly as possible, to be either healthy or sick” (Neumann 1860: 53–54).

One key to success involved giving the proceedings an “air of friendly conversation” (56) that avoided any suggestion of an interrogation. Furthermore, no one could be allowed to interrupt the expert’s presentation, lest the person queried be distracted “just as he was about to reveal the essence of his madness” (57). Neumann also drafted thirteen general rules to guide the questioning in a way that would successfully elicit convincing signs of madness and convince presiding judges (Table 1). Well aware of how precarious these examinations could be, he insisted on the need to call psychiatric experts and rely on their science. For only psychiatrists were familiar with the actual “somatic signs” and “main physiognomic characteristics of insanity” (66, 63). Psychiatrists would also be able to deal more effectively with frenetic patients, silent ones, or those manifesting only periodic or intermittent symptoms, like monomaniacs.

**Table 1:** Allgemeine Regeln [für Gemütszustandsuntersuchungen] nach Heinrich Neumann (1860), *Die Theorie und Praxis der Blödsinnigkeitserklärung nach preußischem Gesetze: Ein Leitfaden für Aerzte und Juristen*, Erlangen: Ferdinand Enke, 58–60.

1	Die erste Frage, überhaupt der Beginn des Gesprächs sei möglichst gleichgültiger Natur und vermeide man den Wahnvorstellungen, überhaupt der besondern Art des Irreseins, gleich nahe zu treten.
2	Man lasse dem Provocaten Zeit zur Antwort; man lasse auch die Fragen nicht Schlag auf Schlag folgen. Die Mehrzahl solcher Individuen bedarf Zeit zur Antwort. Wenn die Antwort schon gegeben ist, und man läßt ihnen etwas Zeit, so fügen sie von der eigenen Ideenassociation geleitet, oft noch einen Nachtrag hinzu. Dieser Nachtrag, der meist einen interessanten Blick in den Ideengang thun läßt, ist daher nicht selten von größerer Bedeutung als der Beginn der Antwort.
3	[die Fragen müssen sich …] immer so genau wie möglich an die zuletzt gegebene Antwort anknüpfen; alles Hin- und Herspringen der Gedanken ist zu vermeiden. Es kostet Zeit und führt vom Ziele ab […].
4	Kommt auch der Kranke nicht bald auf seine falschen Vorstellungen oder bizarren Handlungen zu sprechen, so warte man geduldig. Dies ist keine Zeitverlust, da sich immerhin dabei ein klares Bild von der Persönlichkeit des Kranken, seinem Bildungsgrade, seinem Urtheil, selbst von seinem Charakter entwickeln mag und das gehört recht eigentlich zur Sache.
5	Suggestivfragen vermeide man; sie sind der Nothbehelf ungeduldiger und erfindungsarmer Köpfe.
6	Die erste Erwähnung einer Wahnvorstellung oder einer aus ihr hervorgehenden Handlung giebt dem Gespräche sogleich eine bestimmte Richtung. Jetzt gilt es die einmal aufgefundene Spur nicht wieder zu verlieren, vielmehr den Kranken zu veranlasen, daß er sich möglichst vollständig und ausführlich äußere. Meistens hat dies keine großen Schwierigkeiten, da dem Menschen sein Wahn immer das Wichtigste ist und er sich gern darüber am liebsten vernehmen läßt.
7	Man setze nun das Gespräch so lange fort, bis man glaubt, dem Richter hinlängliche Data an die Hand gegeben zu haben, um das vom Arzte abzugebende Gutachten zu verstehen.
8	Hat der erste Arzt sein Colloquium vollständig beendet, so beginne der zweite, falls er noch irgend einen Punkt für nicht genügend erörtert hält. Ist dies Letztere nicht der Fall, so begnüge er sich mit dem Resultate der eben beendigten Unterhaltung. Denn es kommt überall nicht darauf an, *wer* den Thatbestand erhebt, sondern *daß* und *wie* er erhoben wird.
9	Es ist von der äußersten Wichtigkeit, daß man in denjenigen Fällen, in welchen man sich für das Vorhandensein des Wahnsinns erklärt, sein Augenmerk vorzüglich auf die Vorstellungen, Gefühle, Deutungen dieser Gefühle usw richtet, die Handlungen aber nur insofern berücksichtigt, als sie in jenen Vorstellungen ihre *Motive* haben.
10	Will man hingegen sein Endurtheil auf Blödsinn abgeben, so muß das Hauptaugenmerk auf die Handlungen gerichtet werden, und zwar nicht insofern sie Motive haben, sondern insofern sie Folgen hervorrufen, deren Ueberlegung eben gemangelt hat. Die beiden letzten Regeln sind nicht durch die Natur der Krankheit, folglich auch nicht durch die Wissenschaft, sondern durch die formelle Fassung des Gesetzes geboten.
11	Wird der Kranke gereizt oder gar heftig, so muß man durch die größte Ruhe und oft durch eine geschickte Bemerkung ihn wieder zu besänftigen suchen, denn sonst ist es zuweilen um die Fortsetzung des Termins geschehen.
12	Begegnet etwas der Art, nachdem der Provocat sich schon genugsam ausgesprochen hat, so mag man seiner Heftigkeit etwas Spielraum lassen; die Leichtigkeit, mit der er aufflackert, der Grad der Heftigkeit, zu dem er sich schnell hinaufschraubt, sind dann additionelle Beweise für den veränderten Seelenzustand.
13	Leidenschaftliche Scenen absichtlich hervorzurufen ist gefährlich, inhuman und überflüssig.

The courtroom performance was captured in writing in the court transcript.^11^ But this document was not intended to mirror that performance. On the contrary, the transcript was not a “daguerreotype or mirror image of the interdictee, but rather his … ideal.” It ignored “everything that he had in common with humanity” and depicted instead his specific “peculiarity.” Strikingly, the transcript effectively dispatched the interdictee from the proceedings: “As soon as the transcript is finished, the actual interdictee disappears for the judge and the expert. He is replaced by the files and the transcript. The doctor’s assessment and the judge’s decision are based on these documents, not on the interdictee” (68).

What ensued was then an “entirely abstract” process that had no interest in “reality.” Although a verbatim account of the proceedings was sometimes possible, in many cases the court reporter faced insurmountable obstacles that required him to selectively record the proceedings. The result could be that the case file contained “entirely disparate” documents that “proved nothing” and were of no use to the conscientious expert when drafting his written assessment (70). In practice, these difficulties had led courts simply to rely on memory and the notes made during the “hours long conversation with madmen” before then retrospectively producing a “more agreeable, cogent, and interesting” transcript, albeit an “artificial” one lacking “objectivity” (71).

Prior to German unification in 1871, Neumann was not alone in criticizing these procedural arrangements (Mittenzweig 1888).^12^ Another critic was Wilhelm Sander, who in 1865 was a young assistant physician working on the psychiatric ward of Berlin’s preeminent Charité hospital. He would later go on to serve for decades as the director of Berlin’s first municipal asylum in Dalldorf and was, as such, involved in countless interdiction cases. Sander argued that courts had failed in their responsibility to protect the individual rights of psychiatric patients. He called jurists to account for their lax implementation of existing statutes: “many patients remain in the asylums for years without a court inquiry, as long as they don’t need a guardian; and others still remain in the asylum, even if a court fails to find them demented” (Sander 1865: 201). And to make matters worse, state officials were themselves well aware of these failings and willfully accepted them.^13^

Sander went on to stress that the “exploration in person” represented a threat to patients’ well-being. Many patients became distressed “if urged to speak at length about their condition, to reveal their innermost … feelings and thoughts or to recall uncomfortable memories in front of a stranger” (Sander 1865: 233). Patients were sometimes also fully aware of what was at stake in the proceedings and could suppress or dissimulate their own symptoms. The exploration could thus produce false results and indeed render the entire process meaningless. And finally, because civil proceedings were adversarial and usually initiated by relatives, they had the potential to wreak havoc on patients’ family relations.

One of the professional strategies used to address this dilemma involved making no forensic distinction whatsoever between different forms of insanity. The rallying cry of reformers like Neumann (1860: 93), a prominent advocate of the psychiatric doctrine of unitary psychosis, was: “Classes be gone! Definitions be gone!” And in subsequent decades, in an era of burgeoning specialization and the profuse proliferation of specific psychiatric diseases, forensic experts in Berlin were calling for all relevant cases to be treated simply as mentally ill (Mittenzweig 1888: 225–26).

### Post German Unification: Civil Procedure Code (ZPO, 1877)

Following German unification, efforts to rectify these shortcomings focused on new Imperial legislation, especially the Civil Procedure Code (ZPO) of 1877 (Mendel 1873, 1874). The new ZPO represented a compromise that, above all, aimed to simplify and expedite existing non-adversarial procedures, while simultaneously organizing appellate proceedings (all the way up to the Supreme Court) under the procedural rules of civil courts (Zinn & Pelman 1893: 76). Cases were henceforth initially adjudicated in a so-called *Beschlußverfahren*, with a judge issuing an order based mainly on an evaluation of case files and with no adversarial oral proceedings. As far as the evaluation was concerned, it became first and foremost the responsibility of district courts, where judges were required to examine interdictees in the presence of a court clerk and one or more medical experts (Daude 1882: 52). Although this “personal interrogation” became standard practice, the ZPO also allowed for exceptions if the interdictee was frenetic, severely demented, or at risk of harm because of the examination.^14^ In all cases, however, judges had to justify these exceptions and were furthermore required (unlike psychiatric evaluations in criminal law!) *always* to hear a medical expert before ordering any interdiction.^15^ The examination was almost always held in the residence of the interdictee, often in an asylum (Zinn & Pelman 1893: 73). All proceedings, including the examination, were closed to the public.^16^ Hence, like numerous other practices associated with the study, management, and care of institutionalized populations, the *Gemütszustandsuntersuchung* occupied a (pre-trial) procedural space veiled from public scrutiny.^17^

Perhaps nothing elicited more ongoing and heated debate than the question of exactly which experts needed to be called to help adjudicate cases. Certainly, in parliamentary deliberations on the new ZPO, there had been general agreement that the expert called had to be a medical one. But existing statutes were silent about just what kind of medical expert was required. Alienists (asylum doctors) cited their experience diagnosing mental illness to buttress their claims to forensic legitimacy. District medical officers (*Kreisphysiki*) argued that alienists (especially owners of private asylums), if allowed to evaluate the condition of their own hospital patients, would undercut public faith in court rulings. Forensic medical experts (*Gerichtsärzte*) in turn maintained that familiarity with existing statutes and courtroom procedures was an essential precondition for expert opinions to be heard and heeded. They contended that “one can prepare excellent microscopic slides of the brain, or arrange fine electric baths for nervous patients … yet still be unable to produce a competent forensic assessment” (Mittenzweig 1888: 233). Unlike most other Prussian jurisdictions, Berlin’s courts had their own *Gerichtsärzte*, who were frequently used for interdiction cases.^18^ By contrast, district medical officials were no longer used by the courts after the early 1880s (Pistor 1884: 206).^19^ Berlin’s courts were thus distinctive in relying overwhelmingly on *Gerichtsärzte* and alienists.

Although favorably received in the local liberal press, the reforms did little to assuage critics of interdiction procedures. By the early 1890s, concerns about psychiatric evaluations being used to institutionalize citizens and curtail their rights was blossoming into a nationwide protest movement.^20^ Initially, that movement involved well-to-do patients or their relatives who were mainly concerned about family members being incarcerated in private asylums, which were often run or supervised by Jewish physicians. Psychiatrists and district attorneys throughout Germany became the targets of intense right-wing attacks in the press—as well as in the Prussian and Imperial parliaments—for purportedly colluding with unscrupulous relatives.^21^ Driving the attacks were a group of influential conservative politicians and Lutheran clerics, including the court chaplain and anti-Semite, Adolf Stöcker. The attacks zeroed in on alleged abuses of judicial interdiction—be it to rid families of troublesome members or to profit financially from their hospitalization—thereby helping to fuel a larger “crisis of legitimacy” in German legal circles (Wilhelm 2010: 323–478; Jahr 2011). The director of Brandenburg’s flagship psychiatric hospital in Eberswalde, August Zinn, responded robustly to Stöcker’s accusations in a lecture to the Association of German Alienists in Frankfurt in 1893. A large part of Zinn’s lecture sought to rebut claims that psychiatrists had aided and abetted the unjustified interdictions imposed on healthy, innocent civilians (Zinn & Pelman 1893: 69–97). The Association subsequently resolved that citizens were more than adequately protected by existing legal provisions and that there was no need to reform the ZPO.

As content as psychiatrists may have been with the status quo, criticism of interdictive jurisprudence spread across the political spectrum in the 1890s, fueled nationally by successive public scandals involving illegal internment in psychiatric asylums. In legal circles, concerns about the overreach of psychiatric expertise intensified the reform efforts (Milferstädt 1896: 304, 312). Of seminal importance was the scandalous case of Hermann Sternberg which drew national headlines in the early 1890s and implicated many of Berlin’s most prominent forensic experts, including Wilhelm Sander, Hugo Mittenzweig, and Emanuel Mendel. Another prominent intervention was launched by a group of conservative jurists and public notables—including both the renowned jurist Rudolf von Jhering and the anti-Semitic historian Heinrich von Treitschke—who proposed establishing special interdiction courts that, instead of relying on medical expertise, would impanel a “commission of independent laymen” (including clergy) to assist judges in their findings.^22^ According to these critics, removing medical experts from the proceedings had become necessary because asylum doctors had effectively hijacked the court transcript, usually being able to dictate its content by steamrolling junior court clerks (Kirchenheim & Reinartz 1895: 31). The interdictee therefore needed instead to be interrogated by the judge in the *absence* of asylum doctors.

The standing of asylum doctors, whom Berlin’s courts “always” called, was especially fraught in these debates (Loewenthal 1903: 492). Certainly, their recourse to the extensive clinical evidence acquired over the course of a patient’s institutionalization could be key to effective forensic practice (Cimbal 1907: 15). But by relying on asylum doctors, judges also risked blurring the lines between expert and witness testimony (Schultze 1903a; Pfausler 1902). Once an asylum doctor was sworn in as a medical expert, all of his past clinical observations—often made long before interdiction proceedings began—could be used for his report. But could he testify as a witness to other, legally important but medically irrelevant issues? Or could his expert testimony be impartial if institutionalized patients believed he was mistreating them? Or if his economic livelihood depended on keeping the beds of his private asylum occupied? Could courts demand access to an asylum’s patient records? Such questions had at times led to “serious disharmonies” between judges and physicians (Schultze 1903a: 1).

Given these pitfalls, alienists were not always eager participants in interdiction proceedings. One contemporary survey of asylum doctors found that they preferred not to become involved for fear of undermining the relationships with their patients (Thomsen 1903: 929). Owners of private asylums were also wary of allowing onto their wards the very same state medical officers who were responsible for inspecting their businesses and who were sometimes even professional rivals (Mittenzweig 1888: 214–15). Furthermore, some asylum doctors lamented that the strict regimentation of hospital life made it difficult to demonstrate whether or not patients met the real-world criterion of being unable to attend to their affairs. The hospital’s own disciplinary regime could sometimes produce responsible, quiescent behavior that evaporated upon discharge. To convince skeptical judges, it was therefore paramount that, during the interrogation, patients be confronted with *all* of their past (mis)behavior. And if sufficient evidence was still lacking, doctors might even have to resort to provisionally discharging patients in order to elicit the recidivist misconduct they needed to sway the judge (Hess 1913: 207). While such tactics could be forensically effective, they were likewise accompanied by therapeutic risks.

Compounding these difficulties, the state’s endorsement of alienists’ expertise had never been especially full-throated. Throughout the nineteenth century, governments had relied mainly on state medical officers rather than psychiatric specialists. As a result, even the government’s general efforts to improve forensic assessments ended up sowing doubts in the minds of psychiatric practitioners by appearing to suggest that judges rely primarily not on alienists, but instead on state medical officers (Körting 1905). The uncertainties sparked years-long debate about who best to call as an expert witness.

Doubts about relying on asylum doctors’ intimate knowledge of their patients spurred some critics to reemphasize the judge’s role in interdiction proceedings. One especially prominent example of this view appeared in an article in the *Preußische Jahrbücher*, Berlin’s most influential organ of national liberal orthodoxy. According to its author, Judge Milferstädt (1896: 313–14, 319), among the circa 8 percent of cases that were deemed questionable, non-psychiatric medical experts would inevitably miss some true cases, and psychiatric experts would sometimes over-diagnose false ones. These failings, according to Milferstädt, had sown public mistrust, vindicated psychiatry’s critics, and justified more active intervention on the part of judges. Specifically, the statutes allowing judges to proceed without any examination of the patient needed to be sharply restricted. Instead of simply following schematic, formal procedures, judges needed to be required to examine each case in person. The use of transcripts and deputy judges, who were inevitably less invested in the case, were no substitutes: “Even if the exchange with the patient could be captured by a phonograph, nothing could substitute for personal impression, least of all a court transcript” (ibid.: 316). It was only on the basis of the judge’s personal interrogation that uncertain cases could be identified and doubts about the medical assessment effectively raised.

## *Bürgerliches Gesetzbuch* (1900) and Revisions to the ZPO (1898)

Responding to many of the issues raised in these debates, governments moved to reorganize and clarify the procedures governing interdictive jurisprudence (Rapmund 1895). Above all, the new German Civil Code (BGB) of 1900 reframed the debate by introducing the terms “mental illness” (*Geisteskrankheit*) and “feeble-mindedness” (*Geistesschwäche*) as grounds for interdiction.^23^ Although the terms were widely lauded as an improvement over the now abandoned concepts of *Wahnsinn* and *Blödsinn*, their legal meanings became the topic of considerable debate and consternation in psychiatric circles.^24^ Such uncertainty prompted the nestor of forensic psychiatry in Berlin, Emanuel Mendel, to oppose the term feeble-minded and criticize that it was nowhere defined in the new Code. While some commentators interpreted feeble-mindedness as a psychiatric disorder or symptom, official commentary on the draft BGB legislation had instead defined it in relation to legal and social norms: the key criterion revolved around the ability to conduct one’s affairs—or in legal parlance one’s *Handlungsfähigkeit*—rather than around some medical definition of a natural condition.^25^ In other words, the new Code abstained from defining the terms as having any difference in nature.^26^ Instead, to be interdicted on grounds of insanity and assigned a guardian, individuals had to manifest simply a mental anomaly that made them incapable of managing *all* of their affairs.^27^ Individuals able to manage *some* of their affairs could be interdicted alternatively on grounds of feeble-mindedness and—if they consented—be placed in care (*Pflegschaft*).^28^ One commentator put the difference bluntly: interdiction on grounds of mental illness was nothing but an “incapacity interdiction [*Geschäftsunfähigkeits-Entmündigung*],” and one on grounds of feeble-mindedness was a “limited capacity interdiction [*Geschäftsbeschränkungs-Entmündigung*]” (Levis 1901: 324). Contemporaries spoke of a “large” vs. “small” interdiction (Ilberg 1912: 111). The new Code effectively subsumed the two groups under the existing statutory categories used to define legal capacity (*Geschäftsfähigkeit*): the mentally ill were deemed the legal equivalent of children under the age of seven, and the feeble-minded the equivalent of juveniles over that age.^29^ As a result, phrases like “mentally ill juveniles” or “feeble-minded children” made no sense in civil law (Schultze 1903b: 17–18).

This distinction between mental illness and feeble-mindedness became the subject of endless debate among forensic experts. The radical shift in legal terminology prompted one legal commentator to warn that, in their reports, medical experts be careful to use the terms *Geisteskrankheit* and *Geistesschwäche* only in their legal sense (ibid.: 13, 19). Carl Moeli, director of Berlin’s municipal asylum, Herzberge, and psychiatric advisor to the Prussian Ministry of Culture, went a step further. Moeli (1908/1910: 283–84) recommended that experts *never* use the terms *Geisteskrankheit* and *Geistesschwäche*, but instead rely on more anodyne terms like *Geistesstörung* or *psychische Erkrankung*, so as to clearly demarcate legal from medical terminology.

Compared to prior practices based on eighteenth-century terminology, the new BGB facilitated above all a differentiation of legal consequences. The introduction of interdiction on grounds of feeble-mindedness added a procedural track that enabled judges to target their decisions with greater precision. Specifically, it opened a path that not only facilitated placing patients in care (*Pflegschaft*), but also expanded patients’ legal rights by requiring their consent to do so. In many cases, this became the less onerous and invasive procedural option to interdiction on grounds of insanity.

At the same time, this procedural track contributed to a kind of terminological de-medicalization. Psychiatrists lamented jurists “abjuring the natural qualities” of mental illness and mocked their “succumbing to popular expressions” (Moeli 1899a: 292, 1899b: 11). They were also quick to point to the new Code’s incompatibility with numerous clinical forms, especially periodic and circular disorders. Indeed, they saw little point in trying to align clinical forms to the categories *geisteskrank* and *geistesschwach* (as earlier alienists like Neumann (1860: 38) had done with *Wahnsinn* and *Blödsinn*), since each case now hinged on specific non-psychiatric circumstances (Moeli 1899b: 15). The marginalization of psychiatric assessments was further compounded by what observers saw as a general preference of judges to opt, in uncertain cases, for the lesser alternative of interdiction on grounds of feeble-mindedness (Landauer 1904: 1058). In dubio pro reo!

All of this cast doubt on whether the adjudication of many cases needed to rely on any medical expertise at all (Moeli 1899b: 11–12). Indeed, as medical experts, psychiatrists were hardly in a position to define, let alone demonstrate in a court of law the extent to which someone’s mental anomaly impinged upon their ability to conduct their affairs (ibid.: 18–20). This looming irrelevance of psychiatric expertise spurred one practitioner simply to incorporate “not attending to one’s affairs” into the existing canon of psychiatric “symptoms” (Moeli 1899a: 291). A sympathetic jurist even thought it necessary to reemphasize the importance of psychiatric expertise in such cases, stressing above all just how interwoven mental illness and feeble-mindedness could be (Landauer 1904).

This new legal framework established by the BGB was further buttressed by revisions to the ZPO, as well as new and comprehensive administrative procedural rules. The revised ZPO (1898) codified earlier decrees, making it harder for judges to waive the personal examination, and allowing them to have interdictees committed to hospital (both public or private) for medical observation and evaluation for up to six weeks.^30^ Additional rules were set out in a new order of the Ministry of Justice in 1899, which sought to clarify the responsibilities of district attorneys and courts.^31^ Among other things, the order forbade any consideration of public safety^32^ and required that experts’ full oral testimony (not just their conclusions) be entered into the record.

## Interdictive Jurisprudence at Berlin’s District Courts

Overall, and in combination with the new BGB, these revisions had palpable ramifications for interdictive jurisprudence in Berlin. Specifically, courts resorted increasingly to interdictions on grounds of feeble-mindedness and alcoholism. As a result, both the absolute number of interdictions on grounds of insanity, as well as the proportion in relation to all other cases of interdiction, began to decline markedly after 1900 (Sommer 1910: 323).

The vast majority of interdiction cases in Berlin never elicited any public scrutiny whatsoever. They were adjudicated silently, partly because almost no one cared about many interdictees,^33^ partly because by statute the interrogation was closed to the public.^34^ District attorneys or their representatives were almost never present at the interrogation. Furthermore, although the judge could allow it, neither relatives nor guardians had any right to be present at the proceedings (Koll 1900: 28). And in fact, relatives and guardians rarely attended (Sander 1865: 232; Zinn & Pelman 1893: 74). Occasionally, when family members or their legal representatives were present, they entered statements into the record.^35^ However, experts could object to the presence of relatives, who were then sometimes summarily dismissed before the interrogation began.^36^ In addition, some psychiatrists viewed the involvement of defense lawyers as having “egregious consequences” for patients’ mental well-being and severely disrupting the proceedings (Hess 1913: 210–11).^37^ Moreover, and just like the interdictee, relatives seem to have been almost always shown the door before the expert presented his final assessment.^38^ Finally, if and when interdiction orders were lifted, courts were obliged to publish their decisions *only* in cases involving profligacy and alcoholism, *not* in those involving insanity.^39^ And so the observation of Benjamin Hett (2004: 5) that “Berlin’s jurists worked in a constant glare of publicity” rings true only for the tiny fraction of cases that were appealed to Berlin’s Superior Court.

Overall, in spite of intermittent public scandal and scrutiny, advocates of the status quo were quick to project a generally harmonious image of the forensic work done at Berlin’s district courts. Unlike other investigative venues (police investigations, psychiatric evaluations in criminal trials), civil evaluations were, as dictated by statute, joint ventures of judges and medical experts. If only by virtue of their simple routineness, nowhere else could one expect the potential for collaboration to be greater. And in fact, one judge praised the relationship between court physicians and asylum doctors as the “best imaginable”: judges had been able to collaborate well with these practitioners thanks to their expertise and deference (Thomsen 1903: 930). For the most part, a conciliatory atmosphere seems to have insulated interdiction proceedings from external influences; the forces of collaborative inertia prevailed. In the words of Carl Moeli (1899b: 45) just before the BGB was enacted, any disagreements were but manifestations of “superficial differences” that wouldn’t disrupt the expanded procedural options that the new laws had made available.

Evidence of this collaborative relationship has been preserved in the holding of the Berlin Municipal Archive in the form of medical reports and transcripts of the evaluations.^40^ While the transcripts provide a window onto the interdiction proceedings at Berlin’s district court, they are also fraught with methodological pitfalls for any historical analysis. For one, the preserved documents comprise not the immediate record taken down by the court reporter, but instead a later, well-scripted, and clean (occasionally typed) version of that record. Furthermore, the documents were severed from court and medical case files, and so we can derive from them very little information about how the cases came before the court, and nothing about how the cases were resolved. And finally, needless to say, any attempt to capture the authentic voices of interdictees based on these documents must grapple with the fact they are but products of an inquisitional regime and that their very existence must be put down to the Prussian government’s efforts to enhance that same regime.

### The *Gemütszustandsuntersuchungen*: Documenting Observational Objects and Discursive Subjects

The interrogation itself comprised one of the most important components of the entire legal process of interdiction. The participants interacted in a polygon of asymmetric relationships. It was presided over by the judge and conducted at his discretion.^41^ Although interdictees were not legally required to submit to the interrogation of their own free will, it was necessary and judges could therefore order it.^42^ In this regard, interdictees were less like criminal defendants (who had a right not to testify) and more like witnesses (who had no such right and could be coerced to testify). Similarly, interdictees were not legally required to submit to medical examinations, although legal commentators argued that this too could be ordered by the judge (Levis 1901: 264–66). Procedurally, the interrogation was considered to be part of discovery. And in terms of the rules of evidence, the interdictee was an “observational object [*Augenscheinobjekt*]” (Levis 1901: 209).^43^ The evidentiary value of interdictees’ statements was deemed very small. And although the statements themselves couldn’t be entirely discounted, the manner in which the statements were made was of the “utmost significance,” whether “quickly or carefully, quietly or loudly, sadly or happily [… also the] accompanying muscular movements, a glance of the eyes, the pallor or blush of the face, the trembling or calmness of the body, slower or more rapid breathing” (ibid.: 211).

The expert’s role in the interrogation involved drawing attention to important points that—thanks to his “special psychological training”—he noticed, but that the judge may have overlooked (ibid.: 220). For Mittenzweig (1888: 213), the immediate challenge was to reveal the patient’s “most secret thoughts” to the judge. Just how difficult this could be was not lost on commentators. Indeed, they well recognized that

“nothing was more difficult than interrogating a person so as to discover everything worth knowing about them, without confusing them with false representations or unjustified interventions, and while still pointing out their errors and preventing them from embarking on long-winded digressions that distracted from the facts” (Levis 1901: 253).

The potentially disruptive and harmful effects of the dialogue were a recurrent theme in the literature. Although experts were called upon to present all the relevant information they had gathered, they needed to do so in a manner that avoided provoking or inflicting harm (Moeli 1908/1910: 265). For example, Emmanuel Mendel (1906: 352) warned that in some cases “the attempt to converse with the patient, the wish to disabuse him of his delusions and demonstrate his mental inferiority, causes pain and harm because it excites him, aggravates the illness, and can impede and even endanger recovery.”

Mendel’s comment harked back to long-standing assumptions among psychiatric experts that a “dialectical method of examination” was inappropriate (Meyer 1870: 427–39). This remained a potent argument and psychiatrists continued to worry that if they were not in charge of the interrogation, judges could up-end their work: “The clumsy questions [of the judge] can thwart the entire interrogation and make it impossible for the physician to submit his evaluation” (Hoche 1901: 292). To some extent, such objections likely also reflected their relatively peripheral role in the interrogation. While they had usually conducted extensive preliminary examinations and would subsequently submit to the record an oral or written report of their findings,^44^ their participation in the interrogation itself was supplementary.

Although difficult to assess, the archival record of proceedings before Berlin’s district courts yields only indirect evidence about the working relationship between judges and medical experts. In most cases, the transcripts suggest that the judge initiated the dialogue and the expert occasionally followed up with an additional question or two. Sometimes the transcripts distinguished between the judge’s questions and the expert’s, but often they didn’t.^45^ Some verbatim examples drawn from proceedings before Berlin’s district courts can help illustrate the interrogative and transcriptive practices used in the proceedings, especially the courts’ frequent recourse to a *Gebärdenprotokoll*:

Mit dem Buchdruckereibesitzer Bloßfeld wurde vergeblich eine Verständigung versucht. Beim Erscheinen der unterzeichneten Gerichtspersonen und des Sachverständigers erhob er seinen Kopf leicht und stieß einen Laut aus. Er schien, als ob er sich über das Erscheinen fremder Personen freute. Er gibt auf keine Frage eine Antwort und scheint völlig verständnislos zu beobachten, was mit ihm vorgeht, während der Sachverständige seinen Körper untersucht.^46^

* * * * *

[Der Kaufmann Walter Milbrodt] stieß, während er wankend und geführt, ins Zimmer trat, beständig unartikulierte Laute aus. Er führte einen aus Wolle gefertigten Hund als Spielzeug mit sich. Der Versuch des Richters und des Sachverständigen, den Milbrodt zu vernehmen, erwies sich als vollständig erfolglos. Er blickt mit blöden Gesichtsausdruck bald vor sich her, bald ins Zimmer umher und ist durch keine Frage zu einem Antwort anzuregen. Er giebt weder Auskunft über seinen Namen noch darüber, was er bei sich habe (den Hund) noch wo der Bleistift sei, mit dem er sich Mitteilungen des Sachverständigen bei dessen Vorbesuch beständig beschäftigte. Er ruft nur in Pausen immer wieder aus: „Das kalte Kloster, ich will das kalte Kloster sehen.“ Die Sprache erscheint dabei undeutlich, lallend. Der Gesammteindruck des M. ist der eines Blödsinnigen. Nachdem im Einverständnis mit dem Sachverständiger weitere Versuche der Vernehmung als aussichtslos aufgegeben waren, wurde Milbrodt, der übrigens beständig aus dem Zimmer hinausdrängte, abgeführt …^47^

* * * * *

Sehen Sie ihre Kinder manchmal?

   Nein. Ich möchte meine Kinder ganz gerne sehen, aber nicht hier.

Warum nicht hier?

   Wer in diese Anstalt kommt, der ist in die Hände der Anarchisten und Juden gefallen.

Was tun Sie hier?

   Ich schreibe jetzt seit 2 Jahren.

Was schreiben sie?

   Was der Regierung und der Kirche nützen kann.

Wissen Sie, was das hier für eine Anstalt ist?

   Das weiß ich nicht. Es kann ein Kloster, eine Nervenheilanstalt oder eine politische Anstalt sein.

Fragen des Arztes:

Wie urteilen Sie über das hiesige Pflegepersonal?

   Die Leute hier sind indiszipliniert frech und ungezogen. Sie mischen sich in alles ein, was sie nichts angeht. Sie sind Anarchisten.

Und wie denken Sie über die Bertha?

   Sie tut alles, um meine Verbindungen zu zerstören, Sie leistet Leuten Handgriffe die religiöse Verbindungen durch Jahrtausende haben. Wenn die Juden mir Hunderttausende zur Verfügung stellen u. s. w.

   Wie fing ich doch meinen Satz an? Ich habe den Anfang des Satzes vergessen. Ich bin riguros gegen die Juden, weil sie ihre Verpflichtungen nicht vornehmen, sondern in kleinlicher Weise erfüllen.

Was haben sie noch über die Wärterinnen zu sagen?

   Die Wärterinnen hier haben den Bildungsgrad von Berlin N. Ihr Horizont ist der nächste Laternen-Pfahl.

Als hierauf das Gespräch abgebrochen werden sollte, rief die zu Entmündigende:

   Einen Augenblick Herr Amtsrichter: Ich will Ihnen noch sagen, wie ich mein Geld zu verteilen gedenke: Ein Viertel meines Vermögens soll der Papst bekommen, 1/3 die evangelische Kirche, 1/8 die Juden, 1 Million bestimme ich für die kaiserliche Privatschule, eine weitere Million für jedes Armeekorps und ebensoviel für die Sozialdemokraten.

Die Unterredung wurde hierauf abgebrochen.^48^

### Transcriptive Challenges

One of the remarkable impressions of these transcripts is just how unscripted they appear to be. In contrast to the often boilerplate phraseology used in medical and postmortem reports, the interrogation transcripts were surprisingly open-ended. There were no binding statutory rules governing their content (Milferstädt 1896: 306), although they were required to adhere to general standards of court reporting.^49^ In the contemporary literature one finds no general guidelines for the conduct of medical experts in interdiction cases. In fact, when some reformers called for a formal code of conduct, they were met with skepticism.^50^ The head of Berlin’s Unterrichtsanstalt für Staatsarzneikunde and the founder of the Berlin’s Forensisch-Medizinische Vereinigung, Fritz Strassmann (1908: 1), mocked efforts to develop any kind of “*gerichtsärztliche Knigge*”: no forensic “codex” could ever substitute for the “[instinctive] tact” required of the expert. Likewise for court reporters, for whom it was “tact and understanding” rather than any specific professional skills that were required to successfully fulfill their task (Daude 1882: 55).

The difficulties facing court reporters were self-evident. The interdictee’s responses were sometimes too fast and/or incoherent to be recorded. And conversely, it was sometimes precisely the transcription practices themselves that so agitated interdictees that the interrogation had to be terminated.^51^ The result was often not just an incomplete record with extensive omissions, but a record that could be unwittingly sanitized by the court reporter for the sake of better comprehension. In an article on court reporting practices, the psychiatrist Robert Sommer articulated some of these challenges. Sommer (1907: 212) claimed that too often the transcript’s format didn’t reflect the “mental disposition of the person”; feeble-minded individuals “would have a lengthy and articulate speech attributed to them” that merely “summarized” their statements; the questions were not always “fixed” or free of “suggestive content”; and the recorded response usually failed to capture “pauses, corrections, physiological signs.”

Such transcriptive failings called for compensatory measures.^52^ Upon conclusion of the interrogation, the court reporter was required to show participants the transcript for them to inspect, although if its contents posed a danger to interdictees, it didn’t need to be shown to them (Levis 1901: 223). At this juncture, judges and experts worked to actively craft the transcript. Leppmann (1890: 167) reported that in his experience judges would have court reporters enter into the record “only what the judge, in his own words, considered worthwhile about the personal interrogation, instead of entering the actual dialogue.” And because Carl Moeli (1908/1910: 268) believed that judges’ questions in foro could distract from the aims of the medical expert, he insisted that experts’ questions be explicitly designated as such in the transcript. Failing the use of stenography, he also recommended that elisions be explicitly noted and explained, and that the expert himself take notes on the most important statements and ensure that they were recorded.

## The Debate on Lifting Interdiction Orders

Anyone seeking to challenge interdiction orders—and only the interdictee, not the original petitioner could do so^53^—had two options. They could either appeal their case to the superior courts, arguing that the original order was unjustified (*Anfechtung*), or they could petition the district court that the original conditions justifying the order no longer pertained (*Wiederaufhebung*). In both cases, a new civil case had to be launched, including a new evaluation.^54^ Contemporary observers suggested that because of the “cumbersome … and very costly” adversarial appeals process, the number of lifted interdiction orders never rose substantially.^55^ But when interdiction orders were appealed, cases sometimes spiraled out of control.^56^

On the whole, psychiatrists in Berlin adopted a skeptical attitude toward the lifting of interdiction orders. Mendel for one cited past experience in recommending that experts avoid the term “mentally healthy” in favor of “no longer mentally ill”; and another district court physician maintained that no interdictee should have their rights restored if they were still mentally ill (Mittenzweig 1888: 182, 232). And hard-line skeptics like Arthur Leppmann (1890: 193) insisted that “of the hundreds I helped interdict over the past decade, barely 1 percent could be called healthy.” For him, the essential precondition for any interdiction to be lifted was the patient’s awareness and unequivocal admission of their mental illness. Echoing entrenched mid-nineteenth convictions that many patients (dis-)simulated their illnesses, he warned his colleagues to beware of

“the chronically insane, who over time become so adept at controlling their delusions that nothing serious is revealed even after weeks and months of superficial interaction with them. Especially in these cases, self-awareness of one’s illness is key. If the object [*Explorat*] glosses over his symptoms or tries to explain them away as natural [behavior], this is enough to doubt that his ability to tend to his own affairs has been restored.”

Far from seeking to restrict the realm of interdictive justice, most psychiatrists appear to have been more prone to expanding it, not least to protect other social goods. Addressing the annual meeting of the Association of German Medical Officials in 1913, Leppmann warned that revoking interdictions posed a danger to patients’ relatives, to the general public, and even to medical experts who found themselves targeted by litigious querulants (Leppmann & Hahn 1913: 18–19). Others believed that interdiction could help address security concerns raised by the release of habitual criminals from jail (Dannemann 1912; Hess 1913; Peukert 1983: 75).^57^ Yet such appeals fell on deaf ears, in good part because civil law judges were more inclined to address private interests than public ones (Hartmann 1912: 783).

There were also questions of legal liability that prompted psychiatrists to welcome more frequent interdictions. For as long as hospitalized patients were *not* interdicted and assigned a guardian, Berlin’s asylum directors bore legal responsibility for their physical and financial (!) well-being (Reglement 1900: § 11). Preferring to see a wider swath of their patients interdicted thus reflected rising concerns about patients’ rights and the potential liabilities involved in detaining them against their will. Acting on these concerns, a commission of the German Association for Psychiatry proposed the creation of a legal aid office in Berlin (Siemens 1910).^58^ This suggests that, at least as far as interdictive jurisprudence was concerned, psychiatry was hardly encroaching upon, or undermining legal norms, as is often argued in studies about legal capacity in criminal law. On the contrary: in this instance, an analysis that envisions judicial encroachment on psychiatric practices seems to provide a more plausible narrative.

## Conclusion: The Politics of Interdictive Jurisprudence

The new procedural framework created by the BGB appeared to have taken psychiatrists a step closer to achieving one of their long-standing forensic goals. They came to see feeble-mindedness as effectively “a kind of reduced accountability” in civil law (Cramer 1899: 6). This determination encouraged them to exploit feeble-mindedness in order to advance their larger forensic agendas, specifically to overcome jurists’ objections to the concept of reduced accountability in criminal law, where psychiatrists and reform jurists had long sought legal recognition of reduced accountability, albeit for naught. Some reformers hoped that the BGB’s adoption of the criterion of feeble-mindedness could now allow them to shift cases into civil interdiction proceedings and thereby effectively achieve their long-thwarted forensic aims. In debates at the annual meeting of the Association of German Psychiatrists in 1911, Robert Sommer remarked pointedly: “Interdiction of the criminally feeble-minded is a far better socio-prophylactic measure than mild punishment, with psychiatric implications, on grounds of reduced accountability.”^59^

And yet what I’ve tried to demonstrate is that, at least in Berlin, Sommer’s wish hardly entailed an expansion of psychiatry’s control over interdictive jurisprudence. On the contrary: although psychiatric influence may well have been spreading across other domains, in interdiction cases before Berlin’s district courts it wasn’t. For the most part, psychiatrists failed in their efforts to forensically capture feeble-mindedness. This finding confirms the claim that judicial institutions did not simply “yield” to medical authority (Foucault 1978: 17) and complements a number of other recent studies that have likewise explored the limitations of psychiatric influence (Elrod 2022: 63–64; Balcar 2018: 156, 305; Garz 2022: 217; Engstrom 2022: 87–88).

Was the declining influence of medical expertise in interdiction proceedings a boon for forensic psychology? For potentially, the interrogation of interdictees represented fertile, and as yet largely uncultivated terrain for the introduction of new scientific methods. Certainly, in the years leading up to World War I, Berlin was teeming with recommendations about how to reform forensic work. One lawyer called for an Enquête that could compile and statistically evaluate a “massive collection of precise court transcripts” (Görres 1907). Others reiterated the need for more practical psycho-forensic training in law school and continuing education courses. Another proposed an interdisciplinary *Institut für gerichtliche Seelenkunde* where researchers could demonstrate and experiment on “living material” (Reichel 1910). William Stern (1909) and others proposed reforms in procedural law, police interrogation, and transcription practices (use of stenography), as well as the psychological evaluation of witnesses.^60^ Georg Puppe (1904), a lecturer at Berlin’s Unterrichtsanstalt für Staatsarzneikunde, recommended the establishment of “forensic polyclinics” as a public service, providing free advice and reports that could help preempt disruptions in the legal proceedings. One lawyer even called for the creation of an entire new discipline of “Civil Law Psychology” (Reichel 1910: 301).

Berlin’s judges were certainly aware of these initiatives. In a lecture delivered to the Berliner Juristische Gesellschaft in 1910, the speaker noted that civil and guardianship judges were “constantly dealing with psychological questions (interdiction, divorce, interpretation of legal transactions)” (Reichel 1910). And there is certainly evidence that psychological terminology and reasoning were becoming more prevalent in daily legal practice (Sello 1911; Hett 2004: 195; Mülberger 2009: 76–80). But the reception of many of these reform proposals in the forensic community was anything but warm. Early attempts by defense lawyers to apply psychological experiments during judicial proceeding in Berlin’s courts were rebuffed (Sontag 1907).^61^ Warnings about the dangers of deviating from medical orthodoxy abounded throughout forensic psychiatric literature: traditionalists cautioned that relying on psychological evidence and reasoning would inevitably provoke “crossfire” and undermine the medical evidence (Mendel 1906: 342). And according to Carl Moeli (1908/1910: 267), testing for intelligence, although an important part of the prior exploratory visits, was rare during the interrogation.

Some recent historiography has examined the influence of public discourse on Wilhelmine jurisprudence. Hett (2004: 219) has argued that the “real story of criminal justice in late Wilhelmine Germany” was that publicity and scientific expertise worked to open up courtrooms to decisions that jurists might otherwise not have made and that the public demanded.^62^ Wilhelm (2010) has located a crisis in confidence in legal circles which he traces to jurists’ increasingly fraught relations with the general public. Whether the same can be said for interdictive jurisprudence in Berlin seems doubtful. To be sure, there was no shortage of scandalous cases that garnered sensational headlines. But the findings of this study suggest that decision-making in interdiction cases in Berlin was more likely shaped by judicial inertia, ministerial decrees, or changes in civil law than by public pressure or any purported rise of forensic psychology. And the characterization of the culture of the Wilhelmine courtroom as overly concerned with honor or publicity seems barely to resonate within the routine daily churn of allocating risk at Berlin’s *Amtsgerichte*.

## Footnotes

1 To cite but a few notable examples from a very long list, see Wolffram (2018); Wetzell (2009); Six (2007); Müller (2004); Germann (2004).

2 To again cite but a few prominent examples, see Fischer-Homberger (1975); Lerner (2003); Hofer (2004); Neuner (2011).

3 “Interdiction on grounds of insanity is an act of sovereign assistance [*obrigkeitlicher Fürsorge*] to protect the interests of the patient and his family” (Zinn & Pelman 1893: 84).

4 See Daude (1882); Leppmann (1890); Koll (1900); Kornfeld (1901); Levis (1901, 1906); Moeli (1908/1910).

5 For the purposes of this article, I will be using the term interrogation (*Vernehmung*) in its literal and generic sense of formal questioning. Niehaus (2003: 283) has noted that in criminal procedural law the term *Verhör* largely disappeared in the nineteenth century to be replaced by the term *Vernehmung*. The primary sources on interdiction tend to describe the *Gemütszustandsuntersuchung* using terms such as *Vernehmung, Unterredung, Untersuchung*, or *Exploration*.

6 ALR Thl. I, Tit. 1, §§ 27, 28, 32.

7 For two succinct accounts of the legal process, and actual practice, see Zinn & Pelman (1893: 73–75) and Milferstädt (1896: 304–08).

8 Levis (1901) used the term “interdictees,” which I will do throughout this article as well.

9 AGO (Tit. 38, §§ 1–8), here § 6.

10 See Augustin (1843: vol. 7, 137–38 and 150–52 respectively); Neumann (1860: 43–45); Unger (1898: 22–24); and Daude (1882: 53–55).

11 On the evolution, paradoxes, and pitfalls of the court transcript, see more generally Niehaus (2003: 259–64).

12 More generally on disputes in civil procedural law, see Wilhelm (2010: 251–69).

13 See Sander (1865: 247). To help resolve these problems, Sander recommended that every asylum have a jurist on staff.

14 ZPO (1877) § 598,3.

15 See ZPO (1877) § 599, Daude (1882: 56); Zinn & Pelman (1893: 79–80, 91); Unger (1898: 30).

16 See *Gerichtsverfassungsgesetz* (1877) § 172,2.

17 On the strategies and implications of pre-trial arraignment, see Smith (1981: 170) and Habermas (2008: 138–61). More generally on the varying degrees of public exposure that accompanied different psychiatric practices, and on how that exposure modulated those practices, see Engstrom (2003).

18 In 1900, of seven *Gerichtsärzte* in all of Prussia, four of them worked in Berlin. Administratively, they were part of the police department, but they worked from home (“*Pantoffelbüro*”) and were required to take cases assigned to them by the courts (Pistor 1890: 372–74; Leppmann 1900: 426; Stürzbecher 1997: 64).

19 More generally on the *Stadtphysiki*, see Münch (1995: 92–121).

20 On the political motivations behind their efforts, see especially Engstrom (2017, 2011). For more historical analysis of the broader so-called anti-psychiatry movement, see Schmiedebach (1996) and Goldberg (2002). Beginning in 1891, the Prussian Ministry of Justice carefully tracked newspaper reports about interdictions. See Geheimes Staatsarchiv Preußischer Kulturbesitz (GStAPK) I HA, Rep. 84a, Nr. 5643.

21 Some courts had ruled that Prussian guardianship ordinances gave asylum directors control over patients’ wealth (Zinn 1879).

22 See Engstrom (2011); Siemens & Zinn (1893: 11); and more generally on the push to establish special courts, Wilhelm (2010: 578–95).

23 For accounts of the legal reasoning behind the two concepts and their consequences in law, see Moeli (1899b: 1–18); Levis (1901: 95–106); Schultze (1903b); and Moeli (1908/1910: 272–83). See especially the Supreme Court’s decision of February 13, 1902 (RGZ 50, 203–208—IV 344/01).

24 Some forensic experts understood the new terms as mostly echoing the older concepts of *Blödsinn* and *Wahnsinn* (Cramer 1899: 6; Mendel 1900: 383). Cf. also Peukert (1983: 70).

25 See Moeli (1899b: 6); Cramer (1899: 6); Levis (1901: 61 ff.); and Schultze (1903b: 15).

26 And by the same token, any specific natural cause was of no legal significance (Moeli 1899b: 10).

27 BGB § 6,1.

28 BGB § 1910,2.

29 See BGB §§ 104 and 114.

30 ZPO (1898) §§ 654 and 656. On these provisions, see Levis (1901: 236–49).

31 See the Ministry of Justice’s instructions for interdictions, November 28, 1899, *Justiz-Ministerial-Blatt* (61.44): 388–391, reprinted in Koll (1900: 120–28); Kammergericht Berlin to Ministry of Justice, September 9, 1899, GStAPK I HA, Rep. 84a, Nr. 5642, Bl. 3–4.

32 This stipulation simply reiterated a decision already reached by the Imperial Supreme Court in 1896 (RGZ 38, 191–194—III 183/96).

33 According to Deiters (1902: 30), unlike headline-grabbing criminal cases, interdictions were so routine that they were almost never mentioned in asylum reports.

34 See *Gerichtsverfassungsgesetz* (1877) § 172. Adversarial appellate proceedings, however, were generally open to the public.

35 See the cases of Sczepanski, Haagen, and Zierach, LAB A Pr Br Rep 030 Nr 19776, Bl. 125–137, Nr 19801, Bl. 10–19, and Nr 19802, Bl. 38–62 respectively.

36 See for example the cases of Türpe and Sczepanski, Amtsgericht I, Abt. 81, LAB A Pr Br Rep 030 Nr 19776, Bl. 49 and 125 respectively; also the case of Tänzer, Amtsgericht I, Abt. 81, LAB A Pr Br Rep 030 Nr 19777, Bl. 3.

37 Heather Wolffram (2018: 47) has argued that the professionalization of defense attorneys and their adversarial strategies helped spur the development of forensic psychology. And in fact, it seems that the presence of defense attorneys became more common over time. Cf. the case of Zierach, Amtsgericht Berlin Mitte, Abt. 81, LAB A Pr Br Rep 030 Nr 19802, Bl. 38–62. More generally on disputes about the degree to which civil procedure should be dominated by judges vs. the litigants, see Wilhelm (2010: 578–82).

38 See for example all 12 and 8 cases adjudicated respectively in January 1900 and January 1910, LAB A Pr Br Rep 030 Nr 19777 and 19800.

39 ZPO (1898) § 687.

40 See LAB A Pr Br Rep 030 Nr 19776–19813.

41 ZPO (1898) § 654.

42 See ZPO (1898) § 654 and Levis (1901: 213–14).

43 “During the questioning, the interdictee is, in principle, objective evidence [*sachliches Beweismittel*], an observational object, nothing more than say a dog, about which the judge is trying to conclude whether or not it has been trained as the salesman claims” (Levis 1901: 211).

44 Expert reports were submitted in either oral form, immediately during the interrogation, or in written form. Written reports were submitted later, sometimes only after the expert had been sent the court files or been granted further time for evaluation, and usually only if the case was uncertain and required detailed justification. See Daude (1882: 56–57); Mendel (1906: 353–54); and Moeli (1908/1910: 268).

45 See the cases of Schöpel and Bethmann LAB A Pr Br Rep 030 Nr 19800, Bl. 39–52 and Nr 19802, Bl. 63–76 respectively.

46 Bloßfeld case, Amtsgericht Berlin Wedding, LAB A Pr Br Rep 030 Nr 19801, Bl. 123 f.

47 Milbrodt case, Amtsgericht I, Abt. 83, LAB A Pr Br Rep 030 Nr 19788, Bl. 2–3.

48 Cornelisson case, Amtsgericht Berlin-Schöneberg, Abt. 9, LAB A Pr Br Rep 030 Nr 19802, Bl. 259–266.

49 ZPO (1877) § 145–151; ZPO (1898) § 159–165. The relevant paragraphs remained unchanged between 1877 and 1898.

50 Expert testimony was of course bound by generic procedural rules (ZPO §§ 367–379) as well as the general rules for all witnesses (ZPO §§ 338–366).

51 See the case of Graf Alexander von Döhnhof, Amtsgericht Berlin II, Abt. 1, LAB A Pr Br Rep 030 Nr 19796, Bl. 97–104, specifically Bl. 98.

52 In strictly legal terms, underscoring this need for accuracy in court reporting was the fact that, once the transcript was concluded, its content could be legally contested only as forgery. See ZPO (1877) § 150.

53 See AGO, Titl, 38, §§ 2–9 and Sander (1865: 232).

54 Up until the 1870s, it had been much easier to lift an interdiction than to impose one (Neumann 1860: 45–46). But with the enactment of the ZPO, the procedures for imposing vs. lifting interdictions were made identical. See ZPO (1877) § 604–613 and Zinn & Pelman (1893: 85–87).

55 While the numbers varied slightly, only about 1–1.5 percent of all interdictions were challenged in court, and of those, less than a third were successful. According to Leppmann (1896: 407), appellate courts hardly ever overturned decisions of the district courts. Evidence that so few cases were successfully appealed prompted a government review of the appeals process. See “Auszug aus den Berichten des Oberlandesgerichts-Präsidenten und der Oberstaatsanwaltschaft in Betreff einer Reform des Verfahrens wegen Entmündigung geisteskranker Personen” [1894], GStAPK I HA, Rep. 77, Tit. 298, Nr. 1, Bd. 2.

56 For one spectacular case, see “Zum Fall Rothenburg,” *Vorwärts*, February 11, 1898.

57 In a rhetorical sleight of hand, Hess (1913: 210) called for a change in perspective on interdiction from an “ultimate and permanent elimination of rights” to a “postponement of maturity [*hinausgeschobene Volljährigkeit*].” These suggestions flatly contradicted § 2 of the Ministry of Justice’s order of November 28, 1899, that forbade consideration of public safety in interdiction proceedings.

58 For more on concerns about psychiatrists’ legal liabilities, see Engstrom (2023: 218–22).

59 *Allgemeine Zeitschrift für Psychiatrie* 68 (1911): 536.

60 See also Laurens Schlicht’s contribution in this issue.

61 On the role of defense lawyers in promoting psychological methods in the courtroom, see Wolffram (2018: 47–48). It’s worth noting that the standing of defense lawyers within the Wilhelmine legal system had long remained marginal. Also, a large proportion of the members of Berlin’s bar association were Jewish (Hett 2004: 107).

62 Compare similarly Ortmann (2014: 109–87).
